# Fasting mimicking diet cycles versus a Mediterranean diet and cardiometabolic risk in overweight and obese hypertensive subjects: a randomized clinical trial

**DOI:** 10.1038/s44324-023-00002-1

**Published:** 2023-12-13

**Authors:** Amrendra Mishra, Maura Fanti, Xinzhou Ge, Don Vaughn, Sebastian Brandhorst, Min Wei, Kurt M. Hong, Matteo Pellegrini, Hanno Pijl, Mark C. Houston, Valter D. Longo

**Affiliations:** 1 Longevity Institute, Leonard Davis School of Gerontology and Department of Biological Sciences, University of Southern California, Los Angeles, CA USA; 2 Department of Statistics, University of California, Los Angeles, CA USA; 3 Semel Institute for Neuroscience and Human Behavior, UCLA, Los Angeles, CA USA; 4 Keck School of Medicine, University of Southern California, Los Angeles, CA USA; 5 Molecular, Cell and Developmental Biology, UCLA, Los Angeles, CA USA; 6 Leiden University Medical Center, Department of Internal Medicine, Leiden, The Netherlands; 7 Hypertension Institute, Nashville, TN USA; 8 IFOM, AIRC Institute of Molecular Oncology, Milan, Italy

**Keywords:** Obesity, Metabolism

## Abstract

Abnormalities in the vascular endothelium such as impaired vasodilation can contribute to atherosclerosis and hypertension. Here we have performed a single-center randomized clinical trial to evaluate the efficacy of 4 months of a continuous Mediterranean diet (MD) regimen as compared to 4 cycles of fasting mimicking diet (FMD) administered for only 5 days/month on endothelial function, measured as reactive hyperemia index (RHI) and large/small-resistance artery compliance (AC1/AC2), and on other cardiometabolic risk factors, in hypertensive patients with obesity/excess weight [both sexes, body mass index **(**BMI) ≥ 28, RHI ≤ 2.0, and/or small-resistance artery compliance (AC2) ≤ 5.0]. At the end of the intervention period, FMD but not MD decreased RHI (*p* = 0.0023) compared to baseline with no increase in the portion of patients with abnormal RHI. Both FMD and MD improved PULS cardiac test score; evaluating the risk of cardiovascular events. FMD and MD did not show any significant change in either AC1 or AC2 compared to baseline. Both FMD and MD led to comparable decreases in weight, waist circumference, BMI, body fat mass and % body fat, total cholesterol, and leptin. FMD decreased HbA1c (*p* = 0.0059) and IGF-1 (*p* = 0.0427), while MD decreased glucose (*p* = 0.0488), HOMA-IR (*p* = 0.0476), and HDL-C (*p* = 0.0419). None of the parameters were significantly different between the FMD vs. MD group at the end of the intervention period. During the 3-month follow-up period, the FMD and MD groups continued to display weight and BMI reduction; however, the MD group also lost fat free mass (FMD vs. MD, *p* = 0.0498). In summary, both MD and FMD reduced a range of cardiometabolic risk factors, but FMD also decreased RHI, a change associated with either impaired functional integrity of vascular endothelial cells but also with vascular rejuvenation, with the latter being more likely considering the improved cardiometabolic profile, reduced PULS cardiac score and calculated heart age, and unaltered arterial compliance in the FMD group. MD but not FMD cycles caused loss of lean body mass.

## Introduction

Cardiovascular disease (CVD) remains the leading cause of mortality worldwide^1^. Healthy dietary habits may present an effective strategy to protect the vascular endothelium. As such, the Mediterranean diet (MD; characterized by a high portion of cereals, legumes, vegetables, and fruits, a moderate amount of protein from fish and meat, and fat, especially from olive oil), has been associated with multiple benefits for the prevention of CVD and other diseases^2–5^. MD reduces the incidence of coronary heart disease, stroke, major fatal and non-fatal CVD events^6–9^, and all-cause mortality^5,7,10^, as well as hypertension^11^, improves antioxidant capacity^12^, and decreases total cholesterol^5,13^, oxidized-low density lipoprotein (OxLDL)^4,5^, C-reactive protein^5,13^, and triglycerides^5^ while increasing high-density lipoprotein (HDL) cholesterol^14^. Moreover, MD prevents weight gain^15,16^, and can reduce body mass index (BMI)^17,18^ and waist circumference^14^. Finally, MD improves glucose homeostasis, and insulin resistance^14,16^, decreases the need for antihyperglycemic drugs^19^, reduces the prevalence of metabolic syndrome^14,16,20,21^, and lowers the risk of type 2 diabetes (T2D)^22,23^. However, as a result of the gradual adoption of unhealthy eating habits influenced by Western countries, there has been a surge in the prevalence of obesity and chronic diseases also among the Mediterranean populations over the years^24–26^, indicating that either maintaining or switching to a MD is difficult for the major part of the population. For example, high adherence to the traditional Mediterranean diet decreases from 39.7% in those over 65 to 25.5% of those under 25^27^.

Periodic short-term interventions that do not require long-term changes in dietary habits are emerging as promising strategies in combination with standard treatments for various chronic diseases including CVDs. We have previously shown that a periodic 5-days/month fasting-mimicking diet (FMD) is safe and feasible and improves cardiometabolic risk factors in normal and overweight healthy adults^28^. The FMD is a low-calorie, low-protein, and high fat plant-based diet effective in causing changes in fasting response markers including glucose, ketone bodies, insulin-like growth factor-1 (IGF-1), and insulin-like growth factor binding protein 1 (IGFBP-1) comparable to those induced by water-only fasting^28^.

Here, we conducted a randomized trial to assess the effectiveness of 4 monthly cycles of the Fasting Mimicking Diet (FMD) compared to four months of a continuous Mediterranean Diet (MD) in overweight and obese adults with hypertension.

The primary objective of the study was the assessment of endothelial function determined by the measurement of the reactive hyperemia index (RHI)^29^, and the assessment of large and small resistance arterial compliance (AC1 and AC2, respectively)^30^. RHI, which gives an indication of the endothelial vasodilator function, is inversely correlated with various cardiovascular risk factors^31^. A decrease in peripheral RHI generally suggests impaired functional integrity of vascular endothelial cells, whereas a higher RHI value often indicates the effective compensatory function of endothelial cells^32,33^. Notably, a lower RHI is also associated with younger individuals, indicating that a reduction of RHI can be caused by both vascular impairment but also vascular rejuvenation^34^. Arterial compliance refers to the ability of an artery to expand and contract in response to changes in blood pressure. It is a crucial characteristic of the arterial system that affects blood flow, blood pressure regulation, and overall cardiovascular health. Alterations in arterial compliance correlate with changes in the composition of the arterial wall, such as the ratio of elastic fibers to collagen and the accumulation of disease-related deposits, or changes in muscle tone. A decrease in AC2, indicating poorer small artery compliance, has been observed in subjects with older age, T2D, hypertension, and postmenopausal women with coronary heart disease^35^.

The secondary objectives of the study were represented by changes from baseline to the end of the study in blood pressure, serum lipids, glucose, cardiovascular biomarkers, and body composition.

## Methods

### Study design

This study was designed as a single center, interventional, parallel-group, randomized, open-label, clinical trial aimed at evaluating the effects of four cycles of a once a month 5-day long FMD (provided by L-Nutra inc) in comparison with a continuous regimen of MD for 4 months (Clinicaltrials.gov identifier: NCT04150159).

Different duration of intervention for both FMD and MD have been tested. Most of the initial studies with FMD has been for 3 cycles in 3 months, but recent studies have reported intervention period of 6 (ref. ^36^) or 12 monthly FMD cycles^37^. Mediterranean diet has been tested for range of duration ranging from 28 days to 5 years^38^. The metanalysis by Kastorini et al., 2011, ref. ^38^, also reported that studies with intervention duration of more than 3 months reported significant improvement in most parameters of metabolic health, whereas studies with intervention duration of less than 3 months reported significant improvement in only diastolic blood pressure and glucose^38^. Considering this, an intervention period of 4 months was chosen for this study. The protocol was approved by Salus IRB (2111 W Braker, Austin, TX 78758, USA) and all participants provided written informed consent. None of the sponsors had any role in the study design, data analysis, or reporting of the results.

Clinical visits were performed at baseline (B), 5–8 days after completing the first FMD cycle or 35–38 days after starting the Mediterranean diet (V1), 5–8 days after completing the third FMD cycle or 95–98 days after starting the Mediterranean diet (V2), at the end of the interventional phase: 5–8 days after completing the fourth FMD cycle or 125–128 days after starting the Mediterranean diet cycle/month 4 (V3), and 3-month follow up after the end of the interventional phase (V4). The primary and secondary endpoints of the study focused on evaluating changes in endothelial function and cardiometabolic factors from baseline to the end of the intervention phase (V3).

### Inclusion and exclusion criteria

Subjects of both sexes over the age of 35 to 75 years were recruited in Tennessee, United States, at the Hypertension Institute (HTI) between September 2018 and May 2019. Participants were randomized to the FMD (*n* = 44) or to the MD arm (*n* = 40) using a randomization schedule generated by the clinical staff in blocks of four.

Eligibility criteria included BMI ≥ 28, confirmed diagnosis with either endothelial dysfunction or low small resistance artery compliance (AC2). The endothelial function was assessed noninvasively via peripheral arterial tonometry with the EndoPAT® device. Those with an EndoPAT® score/RHI > 2.0, and/or severe alteration in blood pressure (>180/105) were excluded from the study. The small/large artery compliance (AC1/C2) was measured noninvasively by Computerized Arterial Pulse Wave Analysis (CAWPA) ^30^ using the CV Profiler^TM^ (HDI Hypertension Diagnostics) and defined as AC2 ≤ 5.0 for inclusion.

Exclusion criteria included a history of cardiometabolic disorders (cardiac or cerebrovascular diseases, diabetes mellitus that requires insulin), autoimmune diseases, liver or kidney diseases, any malignancy (unless adequately treated and with no known reoccurrence in the last 2 years), infectious diseases (HIV, TB, hepatitis B or C), serious mental illness, pregnancy, lactating, relevant food allergies, excessive consumption of alcoholic beverages, and drug abuse.

### Outcomes measures

At baseline, information was collected using a lifestyle questionnaire and a personal interview with the doctors. During each visit, data were collected for waist circumference (using an anthropometric tape), body weight (during the bioimpedance analysis, performed via the Body Composition Analyzer BC-418, Tanita), and height (using a wall-mounted stadiometer). Subjects were instructed not to consume caffeine, alcohol, or tobacco products for 6 h preceding (prior to) the blood pressure measurement (measured with a mercury sphygmomanometer, on the left arm, in sitting position, 3 times at 2 min interval, according to the American Heart Association guidelines). Heart rate data was also collected during the blood pressure measurement. The Reactive Hyperemia Index (RHI) was measured at every visit (using the EndoPAT®2000, manufactured by Itamar, Inc). Subjects were given a MicroLife® blood pressure monitor and were instructed on when and how to monitor their blood pressure at home. At every visit, large and small arterial stiffness (AC1 and AC2), along with heart rate, pulse pressure and rate, and blood pressure were measured noninvasively by Computerized Arterial Pulse Wave Analysis (CAWPA; using the CV ProfilerTM, HDI Hypertension Diagnostics) and relative value range was calculated based on each subjects sex and age, according to manufacturer’s instructions (https://www.revolutionhealth.org/cv-profiler-capwa/). Blood samples were collected by venipuncture after an overnight fasting of >12 h between 8 am and 11 am. IGF-1, TNF-alpha, IL-6, and leptin were quantified using standard enzymatic methods and performed by Cleveland Heart labs and Quest Diagnostics. PULS cardiac test (http://www.pulstest.com/) was performed by Predictive Health Diagnostics, 13885 Alton Parkway, Suite B, Irvine CA 92618, United States.

Homeostasis model assessment of insulin resistance (HOMA-IR) was calculated using the following equation: insulin (U/ml) × fasting glucose (mmol/liter) ÷ 22.5. Diabetes prevalence was calculated using the ADA guidelines (HbA1c > 6.4; https://www.diabetes.org/a1c/diagnosis). Metabolic Syndrome prevalence was calculated using the National Cholesterol Education Program Adult Treatment Panel III (NCEP ATP III) definition^39^ as the presence of at least 3 out of 5 of the following metabolic risk factors: waist circumference ≥40 inches for men and ≥35 inches for women, triglycerides ≥150 mg/dL, HDL cholesterol ≤40 mg/dL in men or ≤ 50 mg/dL in women, blood pressure ≥ 130/85 mm Hg, fasting glucose ≥ 100 mg/dL.

### Dietary interventions

The FMD was provided by L-Nutra inc, in a box that contained all the food recommended for the subjects to consume over a period of 5 consecutive days each month. The box consisted of lyophilized vegetable soups, nut bars, and tea bags. The FMD aimed to provide the subjects with an approximate caloric intake of 50–60% of their regular caloric intake on day 1, and 35–40% of their regular caloric intake on days 2 through 5. Assuming the average caloric requirement is 2200 kcal/day for men and 1900 kcal/day for women, the FMD provided a total daily calorie intake ranging from 1000–1100 kcal on day 1 to 700–800 kcal on days 2–5. Most of these calories came from healthy fats (90% on day 1, 40% on days 2–5), while carbohydrates contributed 10–50% and proteins contributed 2–5% of the total calories. The FMD was not adjusted to gender-specific calorie intake, but was adjusted based on person’s weight. Glycerol is given as a daily fasting-associated supplement to serve as a carbon source and help preserve muscle tissue during the period of caloric deficit. In order to promote the safe and efficient utilization of glycerol by patients, the box included guidelines for dosing based on individual body weight.

Both FMD and MD diets provide healthy fats with the difference that the fats in FMD are only plant-based while those in MD recommendations also derived from animal sources. Briefly, fats consumed in the FMD arm come from nuts, seeds, olive oil, and algal oil. They are rich in monounsaturated fats in the form of oleic acid and polyunsaturated fats that include linoleic acid (omega-6) and alpha-linolenic acid (omega-3); docosahexaenoic acid (DHA) and eicosapentaenoic acid (EPA) from algal oil. The main fats in MD components are monounsaturated fats such as oleic acid from olive oil, and polyunsaturated fats such as omega-3 (alpha-linolenic acid) from nuts such, as eicosapentaenoic acid from oily fish.

Participants in the FMD group received a total of 4 boxes, each containing meals for 5 days. They were instructed to consume the entire contents of each box without consuming any additional food. This cycle was repeated every 26 days for a total of 4 months, resulting in 4 FMD cycles. During the periods between FMD cycles, participants were advised to follow their regular diet, and no specific dietary guidelines were provided. On the other hand, participants in the MD group received guidelines and a grocery shopping list based on a validated MD^40,41^. The participants in the MD group were followed by a registered dietitian, but they were not strictly monitored for compliance. Participants on the FMD group were also followed by a registered dietitian during the 5 days of FMD and were advised to only consume the content of the box provided to them.

At the end of the interventional phase, both groups were advised to follow a healthy diet, including fruits, vegetables, whole grains, legumes, nuts, seeds, and heart-healthy fats, and to restrict processed foods, added sugar, and refined grains. Participants in the FMD group were asked to follow this healthy diet and not specifically a Mediterranean diet. Subjects were followed by a registered dietitian during the entire duration of the study.

### Statistics

Data are shown as mean ± SD unless otherwise noted. Normality of the data at baseline was calculated using Shapiro-Wilk test for normality (sample size < 50). Based on these outcomes, baseline characteristics were then compared using either a two-sided unpaired *t*-test (normal distribution) or unpaired Mann–Whitney test (non-normal distribution). We conducted intention-to-treat analysis, which included data from all 84 participants who underwent randomization. Results are reported as intention-to-treat analysis unless indicated otherwise.

For each continuous outcome variable, linear mixed models were used to assess the effect of time and treatment. First, we divided the patients into two groups in terms of treatment (MD/FMD) and used two separate linear mixed models for the two groups to assess the effect of time within each group, with time as fixed effect and patient as random effect. We used the *t*-test to calculate the *p*-value of the coefficient for each of the two groups. Then, we combined these two groups and used another linear mixed model which has time, group, and time*group as fixed effects, and patient as random effect. Group is included in the model to account for baseline differences in the outcome variable between treatment groups. The inclusion of time in the model allows for changes in the outcome over time that are unrelated to the intervention. The interaction terms provide estimates of the treatment effects over time, and the t-test for its coefficient was used to calculate the *p*-value comparing MD with FMD.

For each binary outcome variable, mixed effects logistic models were used to assess the effect of time and treatment. The models and tests are similar to those for continuous outcomes: two separate mixed effects logistic models were used for the two groups to assess the effect of each treatment (time as fixed effect and patient as random effect) and another mixed effects logistic model were used to compare the two treatments (time, group, and time * group as fixed effects and patient as random effect).

For each outcome variable, linear modeling assumptions were assessed with residual diagnostics. The analyses were performed using the R package lme4 and lmerTest.

Samples size was calculated based on previous studies on the FMD^28^.

## Results

### Participants characteristics

**Fig. 1:**
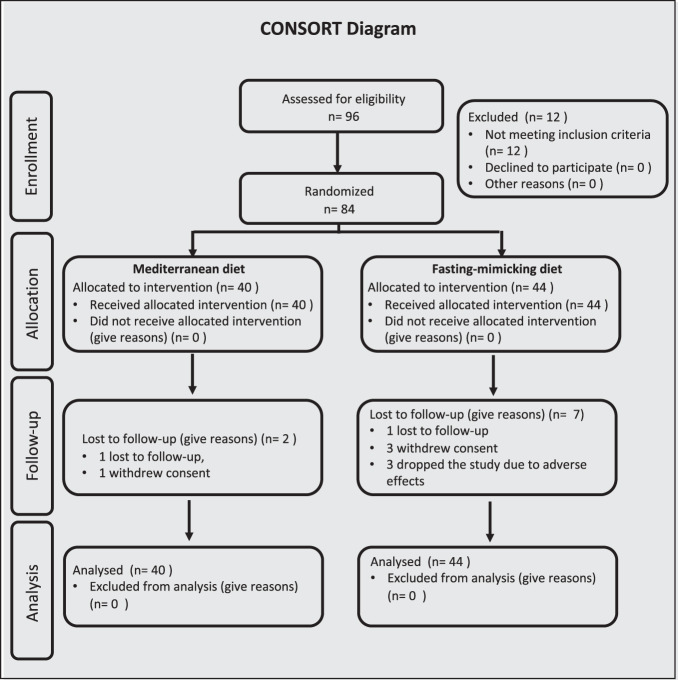
CONSORT Diagram for all stages of the trial.

Among the 96 patients screened, 84 were randomized, and 12 were excluded for not meeting the eligibility criteria. Forty participants were randomized to the Mediterranean diet (MD) and forty-four to the FMD group (Fig. 1). Baseline characteristics in the two arms were similar in terms of BMI, body weight (BW), waist circumference, and blood pressure, with differences in age, sex, race, and body composition (Table 1). The FMD arm included 32 females versus 20 in the MD arm. Out of the 40 patients in the MD arm and 44 in the FMD arm, 26 (65%, MD) and 33 (75%, FMD) received blood pressure medications. During the study, there was little change in the usage of medications (Supplementary Table 1; see Supplemental material). The dropout rate during the intervention phase was 15.9% in the FMD arm [7 of 44] and 5.0% in the MD arm [2 of 40]. Three subjects in the FMD arm dropped out during the first cycle due to adverse events (Supplementary Table 2) with 2 subjects reporting weakness and vomiting, and 1 subject reporting a serious adverse event (patient stated: “*food poisoning due to kale*”; see Supplemental material). Complete baseline characteristics are shown in Table 1.

**Table 1:** Baseline characteristics of the participants.

	FMD	MD	
	mean ± SD (*n*)	mean ± SD (*n*)	*p*-value
General			
Age (years)	54.86 ± 12.38 (44)	63.13 ± 7.72 (40)	**0.0005**
Sex	12 M, 32 F (44)	20 M, 20 F (40)	**0.0434**
Race (C-Caucaccian, B-Black, H-Hispanic)	27 C, 16B, 1H (44)	35 C, 5B, 0H (40)	**0.0221**
Education	10H, 22 C, 12 G (44)	7H, 21 C, 12 G (40)	0.834
Weight (lbs)	213.4 ± 34.94 (44)	221.3 ± 36.51 (40)	0.3133
Waist circumference (inches)	40.16 ± 5.02 (44)	42.15 ± 4.61 (40)	0.0633
BMI (kg/m²)	33.93 ± 4.83 (44)	33.77 ± 5.91 (39)	0.6846
Fat%	40.36 ± 8.14 (44)	36.22 ± 8.95 (39)	**0.0348**
Fat mass (lbs)	86.65 ± 25.03 (44)	80.05 ± 26.20 (39)	0.1601
Trunk fat %	39.34 ± 6.95 (44)	35.95 ± 6.81 (39)	**0.0278**
Trunk fat mass (lbs)	45.39 ± 11.94 (44)	42.46 ± 9.57 (39)	0.224
Fat free mass (lbs)	126.8 ± 24.57 (44)	140.5 ± 29.07 (39)	**0.045**
Sum legs muscle mass (lbs)	40.71 ± 8.32 (44)	45.46 ± 9.93 (39)	0.0559
Cardiac			
RHI	1.95 ± 0.71 (44)	1.75 ± 0.63 (40)	0.1943
Abnormal RHI prevalence	17 (44), 38.6%	18 (40), 45%	0.1887
Heart rate (beats/min)	66.82 ± 11.12 (44)	66.33 ± 9.92 (40)	0.9947
Systolic blood pressure (mmHg)	139.7 ± 18.13 (44)	147.9 ± 17.41 (39)	**0.0406**
Diastolic blood pressure (mmHg)	78.39 ± 9.26 (44)	77.77 ± 8.24 (39)	0.7505
C1 (or AC1) - Large artery elasticity index (ml/mmHg x 10)	12.78 ± 4.33 (44)	11.29 ± 4.29 (40)	0.0867
C2 (or AC2) - Small artery elasticity index (ml/mmHg x 10)	4.63 ± 2.26 (44)	4.033 ± 1.69 (40)	0.2629
PULS score	8.28 ± 7.55 (44)	15.46 ± 13.43 (40)	**0.0017**
Calculated heart age (years)	71.80 ± 12.65 (44)	77.25 ± 6.93 (40)	**0.008**
Heart age difference (years)	16.93 ± 8.89 (44)	14.13 ± 7.76 (40)	0.1023
Serum parameters			
IL6 (pg/ml)	2.69 ± 2.12 (43)	3.71 ± 3.20 (38)	0.0686
Leptin (pg/ml)	15.11 ± 10.09 (43)	16.15 ± 13.71 (39)	0.8192
TNFalpha (pg/ml)	0.96 ± 0.30 (43)	1.12 ± 0.36 (39)	**0.0305**
High-sensitive CRP (mg/dL)	3.78 ± 4.33 (44)	5.18 ± 6.56 (40)	0.4578
Creatinine (urine) (mg/dL)	107.8 ± 70.04 (44)	88.21 ± 54.48 (40)	0.2194
LDL cholesterol (mg/dL)	114.3 ± 40.70 (44)	121.4 ± 33.47 (40)	0.2218
HDL-C value (mg/dL)	54.25 ± 17.12 (44)	52.28 ± 16.47 (40)	0.8463
Triglycerides (mg/dL)	136.5 ± 71.49 (44)	138.7 ± 66.92 (40)	0.6479
Cholesterol-total (mg/dL)	194.1 ± 44.80 (44)	200.0 ± 34.77 (40)	0.2673
Insulin (uU/ml)	15.68 ± 11.65 (44)	18.97 ± 16.56 (40)	0.5944
HbA1c (%)	5.88 ± 0.97 (44)	6.09 ± 0.89 (40)	0.0728
Glucose (mg/dL)	102.5 ± 20.90 (44)	115.5 ± 36.78 (40)	**0.0338**
IGF-1 (ng/ml)	130.2 ± 38.45 (44)	137.5 ± 52.70 (40)	0.7366
MetSyn prevalence	15 (44), 34%	16 (40), 40%	0.6533
Diabetes prevalence	7 (44), 15.9%	12 (40), 30%	0.1911
HOMA-IR	4.15 ± 3.30 (44)	5.66 ± 6.00 (40)	0.3285

Data are shown as mean ± SD unless otherwise noted. Normality of the data at baseline was calculated using Shapiro–Wilk test for normality (sample size < 50). Based on the outcome, baseline characteristics were then compared using either a two-sided unpaired *t-*test (normal distribution) or unpaired Mann–Whitney test (non-normal distribution). We conducted intention-to-treat analysis, which included data from all 84 participants who underwent randomization. Significant values are highlighted in boldface.

*BMI* body mass index, *Waist Circ* waist circumference, *AC1* Large Resistance Artery Compliance, *AC2* Small Resistance Artery Compliance, *RHI* Reactive Hyperemia Index, *H* High school, *C* College, *G* graduate school.

**Table 2:** Results after 4 months of intervention (V3).

	FMD			MD			FMD vs. MD		
	Estimate	Std. Error	*P*-value	Estimate	Std. Error	*P*-value	Estimate	Std. Error	*P*-value
Weight (lbs)	−7.8	1.3	**7.86E-07**	−9.3	1.2	**2.34E-09**	−1.3	1.9	0.487579
Waist circumference (inches)	−1.4	0.4	**0.000526**	−1.9	0.3	**6.25E-08**	−0.4	0.5	0.426845
BMI (kg/m²)	−1.2	0.2	**1.50E-05**	−1.5	0.2	**1.09E-08**	−0.3	0.3	0.33708
Fat%	−2.2	0.6	**0.000665**	−1.8	0.6	**0.003969**	0.5	0.8	0.574188
Fat mass (lbs)	−8.1	1.6	**7.52E-06**	−8.5	1.8	**2.40E-05**	−0.4	2.3	0.87355
Trunk fat %	−1.8	0.6	**0.00232**	−1.5	0.5	**0.009456**	0.3	0.8	0.662362
Trunk fat mass (lbs)	−3.7	0.8	**2.83E-05**	−2.5	1.3	0.065515	1.2	1.5	0.426705
Fat free mass (lbs)	0.1	1.4	0.948786	−1.9	1.4	0.192068	−2.0	2.0	0.312656
Sum legs muscle mass (lbs)	0.5	0.5	0.298557	−0.9	0.7	0.20254	−1.4	0.9	0.105813
RHI	−0.4	0.1	**0.002331**	−0.1	0.1	0.558979	0.3	0.2	0.055801
RHI abnormal prevalence	1.2	0.7	0.066518	0.2	0.6	0.804054	−1.1	0.8	0.193901
Heart rate (beats/min)	−0.4	1.7	0.802074	−2.2	1.5	0.159095	−1.8	2.3	0.439248
Systolic blood pressure (mmHg)	0.9	2.5	0.72006	−3.9	2.4	0.115211	−5.6	3.8	0.139891
Diastolic blood pressure (mmHg)	−0.1	1.3	0.94643	−0.7	1.4	0.598757	−0.7	1.9	0.714982
C1 (or AC1) - Large artery elasticity index (ml/mmHgx10)	−0.5	1.0	0.654825	1.5	0.9	0.1274	1.9	1.4	0.172422
C2 (or AC2) - Small artery elasticity index (ml/mmHgx10)	0.4	0.4	0.30895	0.0	0.3	0.924105	−0.4	0.6	0.46993
PULS score	−0.6	0.6	0.314013	−0.9	1.1	0.396745	−0.3	1.3	0.80391
Calculated heart age (years)	−2.0	1.2	0.107077	−0.6	0.8	0.408835	1.3	1.5	0.387906
Heart age difference	−2.2	1.2	0.066073	−1.0	0.8	0.178256	1.2	1.5	0.432171
MetSyn prevalence	−0.5	0.7	0.425655	0.3	0.8	0.718073	0.9	1.1	0.400982
Diabetes prevalence	−4.5	3.0	0.126174	−14.2	4.4	**0.001232**	−2.1	3.5	0.541908
IL6 (pg/ml)	−0.3	0.3	0.33868	−0.4	0.5	0.442816	−0.1	0.6	0.854638
Leptin (pg/ml)	−4.9	1.1	**9.11E-05**	−5.1	1.0	**5.15E-06**	−0.2	1.5	0.904356
TNFalpha (pg/ml)	0.1	0.0	0.07341	0.3	0.2	0.265535	0.2	0.2	0.461397
High-sensitive CRP (mg/dL)	−1.0	0.7	0.201503	−1.4	1.0	0.149394	−0.4	1.2	0.71252
Creatinine- urine, (mg/dL)	−3.1	13.3	0.817127	15.6	11.1	0.169319	17.7	17.3	0.309356
LDL cholesterol (mg/dL)	−5.7	3.7	0.132536	−5.9	4.7	0.212985	−0.3	6.3	0.965669
HDL cholesterol (mg/dL)	−5.1	2.6	0.053145	−3.0	1.4	**0.041898**	2.4	3.0	0.420084
Triglycerides (mg/dL)	−15.1	8.5	0.085431	−2.1	8.2	0.803328	13.4	12.5	0.2873
Cholesterol-total (mg/dL)	−10.4	4.3	**0.020139**	−10.7	5.0	**0.03778**	−0.4	7.3	0.95635
Insulin (uU/ml)	−1.5	3.2	0.641313	−5.1	2.8	0.075702	−3.6	4.3	0.400295
HbA1c (%)	−0.2	0.1	**0.00593**	−0.2	0.1	0.056449	0.0	0.1	0.786922
IGF-1 (ng/ml)	−9.2	4.4	**0.042703**	−2.2	5.0	0.663645	7.1	6.6	0.286942
HOMA-IR	−0.1	1.2	0.95164	−1.8	0.9	**0.047597**	−1.8	1.5	0.256282
Glucose (mg/dl)	−2.3	3.4	0.508201	−8.9	4.4	**0.048846**	−6.5	5.6	0.244999

For each continuous outcome variable, linear mixed models were used to assess the effect of time and treatment. We used the *t*-test to calculate the *p*-value of the coefficient for each of the two groups. Group is included in the model to account for baseline differences in the outcome variable between treatment groups. The inclusion of time in the model allows for changes in the outcome over time that are unrelated to the intervention. The interaction terms provide estimates of the treatment effects over time, and the *t*-test for its coefficient was used to calculate the *p*-value comparing MD with FMD. Significant values are highlighted in boldface.

*Prevalence at V3 is shown as (%).

*BMI* body mass index (calculated via Bioimpedance as weight/height), *Waist Circ* waist circumference, *AC1* Large Resistance Artery Compliance (measures arterial elasticity), *AC2* Small Resistance Artery Compliance (measures arterial elasticity), *RHI* Reactive Hyperemia Index.

One patient in the MD arm did not have baseline measurements for the BMI and body composition because the scale did not work.

### Diets effectiveness: weight loss and body composition

Changes in body composition from baseline to the end of the interventional phase (V3), during which the FMD group participants had completed four FMD cycles, and the MD group participants 4 months of continuous adherence to MD are shown in Table 2 (linear mixed model analysis).

Both interventions similarly reduced BW (FMD: −7.8 ± 1.3 lbs vs. MD: −9.3 ± 1.2 lbs], waist circumference (FMD: −1.4 ± 0.4inch vs. MD: −1.9 ± 0.3 inch), BMI (FMD: −1.2 ± 0.2 Kg/m^2^ vs. MD: −1.5 ± 0.2 Kg/m^2^), total body fat (FMD: −8.1 ± 1.6 lbs vs. MD: −8.5 ± 1.8 lbs), body fat percentage (FMD: −2.2 ± 0.6% vs. MD: −1.8 ± 0.6%), and trunk fat percentage (FMD: −1.8 ± 0.6% vs. MD: −1.5 ± 0.5%). Only the FMD decreased trunk fat mass (FMD: −3.7 ± 0.8 lbs (*p* < .001) vs. MD: −2.5 ± 1.3 lbs (*p* = .0655)). No other significant differences in body weight and composition were found between the two interventions.

### Primary outcome: endothelial function and arterial compliance

The ability of the arteries to expand and contract with cardiac pulsation and relaxation is called Arterial compliance (AC)^42^. Arterial compliance can be assessed as a function of capacitance of the arterial system measured in larger arteries (AC1), and as a function of the age-dependent reflectance in the arterial system, measured in small arteries (AC2). AC2 correlates with the vascular disease and is associated with hypertension, and atherosclerosis^43^. Both FMD and MD groups showed no change in either AC1 or AC2 at the end of the intervention period. Reactive Hyperemia Index (RHI) is a measure of endothelial function and represents the magnitude of arterial reperfusion following a brief period of occlusion-induced arterial ischemia^29^. RHI decreased in the FMD group (−0.4, *p* = 0.0023) but not in the MD group. However, FMD cycles did not cause an increase in the prevalence of abnormal RHI. There was no difference between the FMD vs. MD group in AC1 (*p* = 0.1724), AC2 (*p* = 0.4699), or RHI (*p* = 0.0558) when compared using the mixed model analysis. Results on the primary outcomes are shown in Table 2.

### Secondary outcome: CMR factors

The secondary endpoint of our study was represented by changes in cardiometabolic risk factors. Changes from baseline to V3 are shown in Table 2 (linear mixed model analysis).

The FMD and MD reduced serum leptin (FMD: −4.9 ± 1.1 pg/ml vs. MD: −5.1 ± 1.0 pg/ml) and total cholesterol level (FMD: −10.4 ± 4.3 mg/ml vs. MD: −10.7 ± 5.0 pg/ml) at the end of the intervention (V3). MD decreased diabetes prevalence (*p* = 0.0012), HDL-C level (*p* = 0.0418), Homeostatic Model Assessment of Insulin Resistance (HOMA-IR; *p* = 0.0475), and serum glucose levels (*p* = 0.0488) at the end of the intervention. Notably, baseline diabetes prevalence in the MD group was 30% versus 15.9% in the FMD group. The FMD cycles decreased HbA1c level (*p* = 0.0059) and IGF-1 level (*p* = 0.0427) at the end of the intervention.

Other parameters did not change significantly in either group at the end of the intervention and none of the parameters were significantly different between the FMD and MD groups at V3 (linear mixed model analysis, Table 2).

### Follow up - result maintenance

Results after a 3-month follow-up (V4) are presented in Table 3.

**Table 3:** Results after 3 months of follow up (V4).

	FMD			MD			FMD vs. MD		
	Estimate	Std. Error	*P*-value	Estimate	Std. Error	*P*-value	Estimate	Std. Error	*P*-value
Weight (lbs)	−5.4	1.9	**0.006196**	−10.4	1.7	**7.02E-07**	−4.7	2.9	0.107374
Waist circumference (inches)	−0.8	0.3	**0.01946**	−1.2	0.3	**0.000618**	−0.3	0.5	0.51075
BMI (kg/m²)	−0.8	0.3	**0.024116**	−1.8	0.3	**1.05E-07**	−1.0	0.4	**0.022766**
Fat%	−1.0	0.6	0.101736	−1.4	0.7	**0.041597**	−0.4	0.9	0.626997
Fat mass (lbs)	−4.6	1.7	**0.012176**	−7.1	1.9	**0.000624**	−2.5	2.5	0.332553
Trunk fat %	−0.7	0.6	0.235738	−1.5	0.7	**0.037634**	−0.7	0.9	0.434705
Trunk fat mass (lbs)	−1.8	0.9	**0.04176**	−3.6	1.1	**0.002947**	−1.7	1.4	0.236341
Fat free mass (lbs)	−0.9	1.5	0.546639	−4.7	1.3	**0.000804**	−3.9	1.9	**0.049867**
Sum legs muscle mass (lbs)	0.0	0.5	0.993048	−2.3	0.6	**0.000456**	−2.3	0.8	**0.003228**
RHI	−0.4	0.2	**0.022259**	0.0	0.1	0.934113	0.4	0.2	**0.040603**
RHI abnormal prevalence	1.0	0.6	0.077676	−0.1	0.6	0.873792	−1.1	0.8	0.133505
Heart rate (beats/min)	−0.3	2.0	0.87432	−2.1	2.3	0.371837	−1.5	3.0	0.613259
Systolic blood pressure (mmHg)	3.6	2.5	0.170322	−1.6	3.5	0.646739	−5.8	4.3	0.187445
Diastolic blood pressure (mmHg)	0.5	1.5	0.736755	−0.9	1.3	0.489266	−1.5	2.0	0.464687
C1 (or AC1) - Large artery elasticity index (ml/mmHgx10)	−0.1	0.8	0.913496	1.7	1.0	0.081858	1.8	1.3	0.157479
C2 (or AC2) - Small artery elasticity index (ml/mmHgx10)	0.3	0.5	0.545177	1.2	0.4	**0.011089**	0.9	0.7	0.220647
PULS score	−1.5	0.6	**0.022492**	−2.3	1.1	**0.039072**	−0.8	1.3	0.526924
Calculated heart age (years)	−2.6	1.5	0.091296	−1.9	1.1	0.090032	0.5	2.0	0.794108
Heart age difference	−3.0	1.5	**0.04558**	−3.0	1.1	**0.012803**	0.0	1.9	0.980291
MetSyn prevalence	0.0	0.7	0.978191	−0.6	0.8	0.417588	−0.6	1.0	0.55771
Diabetes prevalence	−3.0	2.4	0.213434	−14.2	4.4	**0.001274**	−5.0	4.3	0.240175
IL6 (pg/ml)	0.3	0.3	0.274541	0.0	0.5	0.976424	−0.3	0.6	0.599972
Leptin (pg/ml)	−4.6	0.9	**7.52E-06**	−5.7	1.3	**9.12E-05**	−1.1	1.5	0.4823
TNFalpha (pg/ml)	0.1	0.1	0.074772	0.3	0.2	0.21916	0.2	0.3	0.412241
High-sensitive CRP (mg/dL)	0.3	0.4	0.459005	−1.4	1.2	0.261142	−1.3	1.2	0.253096
Creatinine- urine, (mg/dL)	5.8	11.4	0.612214	11.5	11.0	0.301506	5.8	15.9	0.716799
LDL cholesterol (mg/dL)	−3.2	2.7	0.235695	−5.4	4.6	0.246857	−2.3	5.7	0.683612
HDL cholesterol (mg/dL)	−5.0	2.8	0.085307	−0.3	1.4	0.829028	5.1	3.2	0.108866
Triglycerides (mg/dL)	4.2	6.5	0.521597	−14.8	9.1	0.113515	−17.9	13.0	0.171995
Cholesterol-total (mg/dL)	−6.3	3.8	0.105568	−9.3	4.7	0.056773	−2.8	7.0	0.693429
Insulin (uU/ml)	−5.6	1.9	**0.004583**	−2.8	5.0	0.57171	2.8	5.2	0.590103
HbA1c (%)	−0.1	0.1	**0.011585**	−0.2	0.1	0.053807	−0.1	0.1	0.466932
IGF-1 (ng/ml)	−6.9	4.3	0.121432	−7.0	6.0	0.25169	0.0	7.4	0.997548
HOMA-IR	−1.5	0.5	**0.006643**	−1.6	1.4	0.256794	0.0	1.4	0.976463
Glucose (mg/dl)	−0.3	2.1	0.886894	−7.3	6.4	0.258733	−7.2	6.8	0.291929

For each continuous outcome variable, linear mixed models were used to assess the effect of time and treatment. We used the *t*-test to calculate the p-value of the coefficient for each of the two groups. Group is included in the model to account for baseline differences in the outcome variable between treatment groups. The inclusion of time in the model allows for changes in the outcome over time that are unrelated to the intervention. The interaction terms provide estimates of the treatment effects over time, and the *t*-test for its coefficient was used to calculate the *p*-value comparing MD with FMD. Significant values are highlighted in boldface.

Results observed on RHI at V3 were maintained during the follow-up in the FMD group [V4 vs. baseline: FMD −0.4 (*p* = 0.0222), MD 0.0 (*p* = 0.9341); FMD vs. MD− *p* value = 0.0406, linear mixed model analysis, Table 3]. The MD group showed a significant increase in AC2 compared to the baseline (+1.2 ± 0.4, *p* = 0.0110), while the FMD group did not (+0.3 ± 0.5, *p* = 0.5451), with no significant difference between the FMD and MD groups (*p* = 0.2206, linear mixed model analysis).

Both groups maintained the lost weight, BMI, fat mass, and reduced leptin levels after the 3-month follow-up period (Table 3). Compared to baseline, at the end of the follow-up period, the MD group lost a significant amount of FFM [MD: −4.7 ± 1.3 lbs (*p* = 0.0008)] which was not observed in the FMD group [FMD: −0.9 ± 1.5 lbs (*p* = 0.5466); FMD vs. MD, *p* = 0.0498, linear mixed model analysis] and leg muscle mass [V4 vs. baseline; −2.3 lbs (*p* = 0.0005)] which was also not observed in the FMD group [FMD vs. MD, *p* = 0.0032, linear mixed model analysis].

Both FMD and MD reduced PULS (Protein Unstable Lesion Signature) cardiac test scores; a test that evaluates 5-year risk of a heart attack or stroke^44^, after the 3-month follow-up period as compared to the baseline but the PULS score did not differ between groups [V4 vs. baseline; FMD: −1.5 (*p* = 0.0224) vs. MD: −2.3 (*p* = 0.0390); FMD vs. MD, *p* = 0.5269, linear mixed model analysis]. In the same test, both groups also showed a reduced calculated heart age and reduced heart age difference [V4 vs. baseline; FMD: −3.0 years (*p* = 0.0456) vs. MD: −3.0 years (*p* = 0.0128); FMD vs. MD, *p* = 0.9803, linear mixed model analysis]. Additionally, the FMD decreased insulin (−5.6 uU/ml; *p* = 0.0046), HbA1c (−0.1; *p* = 0.0116), and HOMA-IR (−1.5; *p* = 0.0066) levels during the follow-up as compared to the baseline, changes not observed in the MD group.

## Discussion

In this randomized controlled intervention trial, the primary outcome of the study was the evaluation of endothelial function, measured by small and large arterial compliance (AC1/AC2) and reactive hyperemia index (RHI). Neither the FMD nor the MD intervention caused significant improvements in arterial compliance measures. The FMD group exhibited a reduction in reactive hyperemia index (RHI), which could be interpreted as a potential impairment in endothelial function. However, several studies have indicated lower RHI values in younger individuals^34,45^ and no correlation with endothelial function values measured using gold-standard brachial artery “flow-mediated dilation”^45^, in agreement with the results shown here. This is relevant because the 4 FMD cycles caused an average 2.5 years biological age reduction in the patients of this study, as measured by the BioAge method (S.B. et al., unpublished observations). A study by Nilsson, et al. measuring RHI among healthy individuals between the age of 18-30 years reported that 47% of the study participants had RHI below the cutoff of 1.67, again supporting the possibility that RHI may also be low as a result of changes in the heart consistent with rejuvenation^46^. In fact, Jujic et al. also reported that among healthy individuals (total 1812) even the abnormal RHI < 1.67 levels was more common in younger individuals aged ≤30 years (47.4%), compared to older individuals aged ≥30 years (27.6%)^47^. We performed the Spearman’s correlation test on our data at baseline that gave the Spearman’s rank correlation coefficient of -0.2108 (data not shown), signifying a negligible correlation between RHI and age^48^. In addition, neither the FMD nor the MD group showed changes in the prevalence of abnormal RHI (< 1.67)^49^. In summary, the reduction in the PULS cardiac test scores evaluating the 5-year risk of a heart attack or stroke, a trend for reduced calculated heart age after the 3-month follow-up period, along with a reduction in biological age in the patients who were treated with 4 FMD cycles, but also considering the lack of small and large arterial compliance changes it is more likely that a reduced RHI represents a rejuvenating effect of FMD cycles rather than an impairment of endothelial function. However, we do not know the reason for decreased RHI at this stage and further studies are needed to explain the significance of these findings.

Four cycles of a Fasting Mimicking Diet (FMD) provide a range of changes overlapping with but also distinct from those achieved by 4 months of continuous Mediterranean diet (MD) in overweight and obese individuals at risk for cardiovascular disease (CVD). After the 3-month follow-up period, FMD showed a more sustained long-term effects with decreased Insulin, HbA1c, and HOMA-IR level. These results are in line with the positive effects of FMD in patients with type II diabetes or prediabetes reported in previous studies^28,36^. Notably, the MD group showed greater decrease in prevalence of diabetes, although this group had a nearly double portion of subjects with diabetes at baseline. Considering the effect of FMD cycles in causing diabetes regression^36,50^, but also the effects on insulin, HbA1c, and HOMA-IR shown in this trial, the much smaller diabetes portion in the FMD group compared to the MD group is likely to have affected the statistical significance of the changes.

**Fig. 2:**
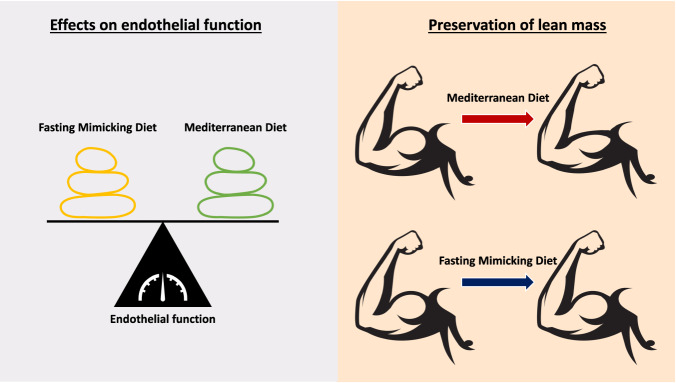
Efficacy of FMD vs. MD for improvements in endothelial function and preservation of lean mass. Both 5-day/month FMD and continuous MD has similar effects on endothelial function during the 4-month intervention period with no difference observed between FMD vs. MD group. MD but not FMD cycles caused loss of lean body mass.

These findings indicate that both interventions can effectively contribute to weight management and improve cardiometabolic disease risk (Fig. 2). Notably, only the FMD intervention showed a significant decrease in trunk fat mass, suggesting a potential advantage in targeting abdominal fat reduction.

Sarcopenia or the loss of lean muscle mass is associated with aging^51^. Notably, while the FMD group showed no change in lean muscle mass, the MD group showed nearly 5 lb loss of FFM and over 2 lb loss of leg muscle mass at the end of the follow-up period, which could contribute to increased frailty, falls and fractures in old age^52^.

With limited consumption of meat, poultry, eggs, and milk, the Mediterranean diet has been reported to provide a 20% lower protein content as compared to the typical American diet^53^. Whereas in some studies the shift from a relatively healthy diet to the Mediterranean diet did not cause lean body mass loss, we speculate that the shift from western style diet to chronic Mediterranean diet in many of the overweight and obese subjects in this study may have caused a reduced protein intake but, more importantly, a major decrease in certain amino acids which promote muscle growth and which are known to be several fold lower in legumes and certain other plant based protein sources compared to animal-derived proteins. FMD on the other hand is a short-term intervention lasting only 5 days a month, which does not interfere with the long-term dietary habit and hence is unlikely to change the overall protein content of the participant’s diet. Loss of lean mass in our study is in line with other studies on Mediterranean diet in obese individuals. For example, Andreoli et al. reported that among obese women, 2 month of a Mediterranean diet lead to significant loss of fat free mass^54^. The significant difference between the FMD and MD groups in this regard suggests that the periodic FMD may be more effective in preserving lean body mass compared to the continuous MD intervention.

The limitation of the study includes the unbalanced distribution of some parameters at the baseline, which could confound the interpretation of the results. These include age, sex, % fat, % trunk fat, fat free mass, and systolic blood pressure, PULS score, calculated heart age and TNFalpha. However, we used mixed effects logistic model to compare the two treatments (time, group, and time * group as fixed effects and patient as random effect) to overcome the limitations of unbalanced groups at baseline. Group is included in the model to account for baseline differences in the outcome variable between treatment groups (see Method section for details). Another limitation was the use of RHI as a marker for evaluating the impact of diets on endothelial function in obesity, instead of Flow Mediated Dilation, the gold standard vascular function test for the measurement of brachial artery dilation^55^, that was not available at the clinical site.

Finally, the study personnel reported that participants who did not enjoy the taste of the FMD food found it challenging to adhere to the monthly FMD cycle. This fact may explain the higher dropout rate in the FMD group. It also suggests that providing more food options and diversifying the FMD menu may improve adherence and meet the needs of individuals who find periodic diets more feasible than continuous dietary changes. Additionally, Jospe et al., 2020 (ref. ^56^), reported that in a 12-month, self-selected dietary intervention study a higher percentage of individuals chose intermittent fasting (54%) as compared to Mediterranean diet (27%) or paleo diet (18%). However, higher adherence was reported by individuals on Mediterranean diet (57%) as compared to the intermittent fasting (54%) and paleo diet (35%)^56^. A higher but mild adverse event has been reported in other fasting trials as well, where TRE showed higher adverse event related dropout compared to that in Mediterranean diet^57^.

In conclusion, both 5-day/month FMD and continuous MD showed improvements in CMR factors during the 4-month intervention period (Fig. 2). FMD cycles showed a long-term decrease in Insulin, HbA1c, and HOMA-IR level that lasted until the 3-month follow-up period, while MD group showed greater decrease in prevalence of diabetes, although the portion of subjects with diabetes was nearly twice as high in the MD versus FMD group at baseline. After the 3 months follow-up MD but not FMD caused lean body mass loss, while FMD decreased RHI, a change associated with impaired integrity of vascular cells but also with younger age, which would be consistent with the reduced biological age measured after FMD cycles (S.B. et al., unpublished observations).

## Supplementary information

Supplementary information

## Data Availability

M.C.H. had full access to the hard copies of the data and is responsible for the integrity of the clinical records and the data source. Z.G., D.V., S.B. and V.D.L. had access to blinded and deidentified data. Deidentified data is available upon request from the corresponding author, V.D.L. Researchers need to provide a methodologically sound proposal and sign a data access agreement to gain access to data. Data is not publicly available to protect and comply with patient privacy/consent.
