# Publisher Correction: Fasting mimicking diet cycles versus a Mediterranean diet and cardiometabolic risk in overweight and obese hypertensive subjects: a randomized clinical trial

**DOI:** 10.1038/s44324-025-00084-z

**Published:** 2025-09-23

**Authors:** Amrendra Mishra, Maura Fanti, Xinzhou Ge, Don Vaughn, Sebastian Brandhorst, Min Wei, Kurt M. Hong, Matteo Pellegrini, Hanno Pijl, Mark C. Houston, Valter D. Longo

**Affiliations:** 1 Longevity Institute, Leonard Davis School of Gerontology and Department of Biological Sciences, University of Southern California, Los Angeles, CA USA; 2 Department of Statistics, University of California, Los Angeles, CA USA; 3 Semel Institute for Neuroscience and Human Behavior, UCLA, Los Angeles, CA USA; 4 Keck School of Medicine, University of Southern California, Los Angeles, CA USA; 5 Molecular, Cell and Developmental Biology, UCLA, Los Angeles, CA USA; 6 Leiden University Medical Center, Department of Internal Medicine, Leiden, The Netherlands; 7 Hypertension Institute, Nashville, TN USA; 8 IFOM, AIRC Institute of Molecular Oncology, Milan, Italy

**Keywords:** Obesity, Metabolism

Correction to: *npj Metabolic Health and Disease* 10.1038/s44324-023-00002-1, published online 13 December 2023

In this article, there are amendments to the Methods, Discussion, Author contributions and Competing interests sections. The original article has been corrected. The following changes were made:

Methods

In the Study design section, the rationale for intervention duration was more fully detailed, with additional citations provided:

“Different duration of intervention for both FMD and MD have been tested. Most of the initial studies with FMD has been for 3 cycles in 3 months, but recent studies have reported intervention period of 6 (ref. 58) or 12 monthly FMD cycles55. Mediterranean diet has been tested for range of duration ranging from 28 days to 5 years^57^. The metanalysis by Kastorini et al., 2011, ref. 57, also reported that studies with intervention duration of more than 3 months reported significant improvement in most parameters of metabolic health, whereas studies with intervention duration of less than 3 months reported significant improvement in only diastolic blood pressure and glucose^57^. Considering this, an intervention period of 4 months was chosen for this study.”

Discussion

In the paragraph starting “Finally, the study personnel reported that participants who did not enjoy the taste of the FMD”, further information about adherence to diets and citations were added:

“Additionally, Jospe et al., 2020 (ref. 56), reported that in a 12-month, self-selected dietary intervention study a higher percentage of individuals chose intermittent fasting (54%) as compared to Mediterranean diet (27%) or paleo diet (18%). However, higher adherence was reported by individuals on Mediterranean diet (57%) as compared to the intermittent fasting (54%) and paleo diet(35%)^56^. A higher but mild adverse event has been reported in other fasting trials as well, where TRE showed higher adverse event related dropout compared to that in Mediterranean diet^54^.”

Author contributions

Missing author contributions were added:

“V.D.L., M.W., M.C.H. and designed the study. M.C.H. conducted the study. X.G., D.V. and A.M. performed data analysis. A.M. and M.F. drafted the manuscript and S.B., V.D.L., K.M.H., M.P., H.P., and all other authors contributed to the critical revision of its content. A.M., D.V. and S.B. performed and verified data analysis. All authors have read and agreed to the published version of the manuscript.”

Competing interests:

The relationship and funding situation between the authors, their affiliations and L-Nutra was clarified:

A grant was provided to the Hypertension Institute in Tennessee by L-Nutra. The Hypertension Institute under the direction of Dr. Mark Houston performed all of the clinical tests in the study. None of the patients were enrolled or studied at USC. The clinical study was sponsored by L-Nutra Inc, which provided the experimental FMD and paid for all the associated procedures and lab tests here presented, making an exception for the PULS test, which was performed at no cost by Predictive Health Diagnostics, 13885 Alton Parkway, Suite B, Irvine CA 92618, United States. The USC-based personnel involved in the study, including the authors of this manuscript, did not receive any compensation from L-Nutra. All the work conducted at USC, data digitalization, as well as the data analysis was verified by an independent statistician at UCLA. This confirmation analysis was funded by a USC chair account under V.D.L. L-Nutra Inc. had no involvement in the study design, collection, analysis, and interpretation of data, writing of the report, manuscript revision or decision to submit the article for publication. V.D.L. who has an equity interest in L-Nutra, did not participate in the collection and analysis of the data. None of the authors that performed data collection or analysis have equity in or are receiving consulting fees from L-Nutra.

V.D.L. has equity interest in L-Nutra and owns the patents of the Fasting Mimicking Diet: US 20140227373A1; US 20140112909A1. V.D.L., S.B., and M.W. hold patents related to the Fasting Mimicking Diet but which are not a result of this trial. The University of Southern California has licensed intellectual property to L-Nutra Inc. As part of this license agreement, the University has the potential to receive royalty payments from L-Nutra Inc.

