# Supplementary material for: Fasting mimicking diet cycles versus a Mediterranean diet and cardiometabolic risk in overweight and obese hypertensive subjects: a randomized clinical trial
Source: NPJ Metab Health Dis. 2023 Dec 13;1:1. doi: 10.1038/s44324-023-00002-1 (PMC12118742; doi:10.1038/s44324-023-00002-1)
Supplement: Supplementary file 1 — Supplementary information [file 44324_2023_2_MOESM1_ESM.docx]

**Supplementary Information**

**Supplementary Table 1. Medication usage**

All subjects enrolled in each arm were included. Intention-to-treat analysis.

Significance was calculated using Fisher's exact test: p < 0.05.

**Supplementary Table 2. Adverse Events**

Detailed description of the adverse events.

|  | **FMD (n=38)** (number of times) | | **MD (n=39)** (number of times) | | **nonparametric Mann-Whitney test** |
| --- | --- | --- | --- | --- | --- |
|  | **mean** | **SD** | **mean** | **SD** | **p-value** |
| **Red meat** (weekly) | 2.211 | 1.298 | 2.282 | 1.503 | 0.9314 |
| **Processed meat** (weekly) | 1.421 | 1.328 | 1.436 | 1.334 | 0.9812 |
| **Fried Food** (weekly) | 1.474 | 1.179 | 2.103 | 1.252 | **0.0199** |
| **Pastries** (weekly) | 1.632 | 1.731 | 1.282 | 1.572 | 0.3443 |
| **Fish** (weekly) | 1.789 | 1.094 | 1.667 | 1.06 | 0.6387 |
| **Vegetables** (weekly) | 4.949 | 1.754 | 5.513 | 1.745 | 0.1419 |
| **Fruits** (weekly) | 3.553 | 2.127 | 4.436 | 2.036 | 0.0754 |
| **Milk products** (weekly) | 2.974 | 2.52 | 3.718 | 2.316 | 0.1633 |
| **no. of meals** (daily) | 2.789 | 0.8107 | 2.692 | 0.6551 | 0.7670 |

**Supplementary Table 3. Participants’ self-reported dietary information collected during intake survey.**

Participants were asked to fill up a questionnaire asking how many times per week they consume red meat, processed meat, fried food, pastries, fish, vegetables, fruits, milk products as well as the daily number of meals. Participants in both groups reported similar weekly consumption of red meat, processed meat, pastries, fish, vegetables, fruits, milk products, and daily number of meals. MD group reported higher consumption of fried food as determined by the nonparametric Mann-Whitney test (non-normally distributed data). The average number of daily meals was <3 for both groups and fruits and vegetables were consumed at a higher frequency than red meat and processed meat.

**Mediterranean diet guidelines handout -page 1**


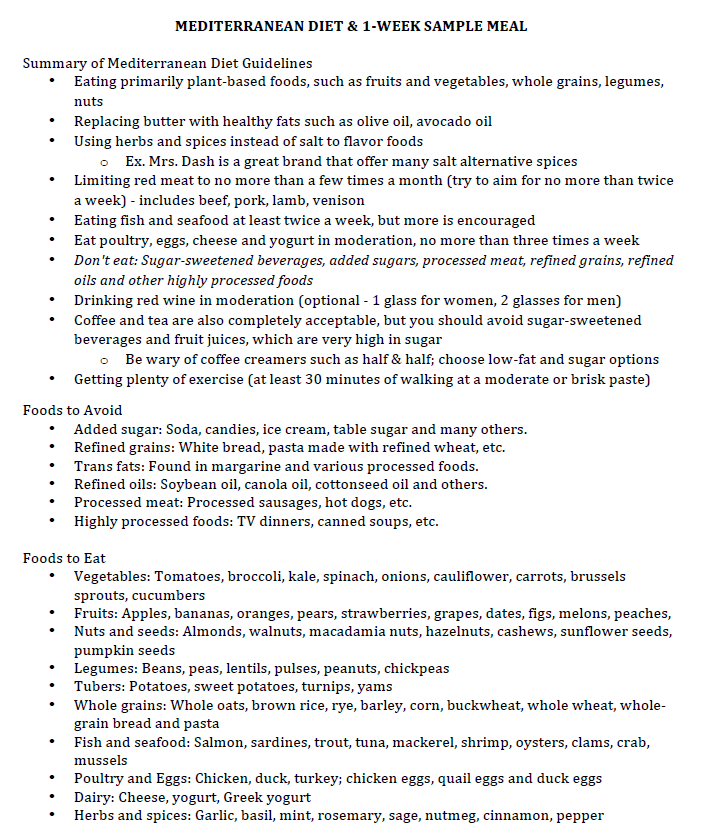


**Mediterranean diet guidelines handout -page 2**


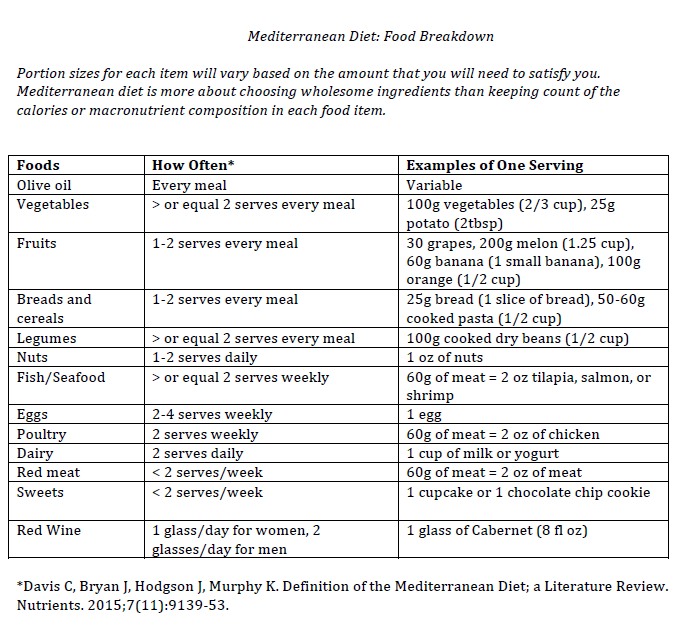


**Mediterranean diet guidelines handout -page**
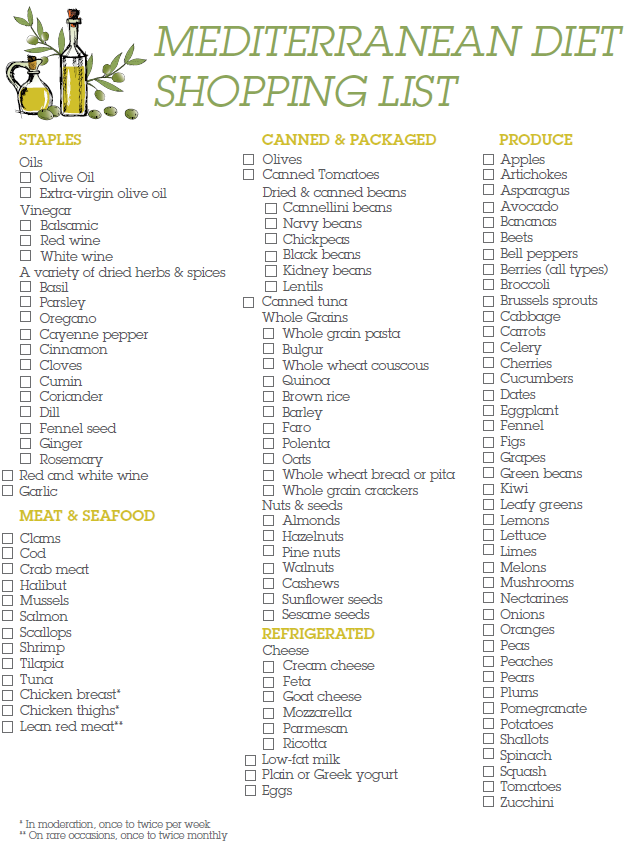
**3**
